# Comparison of a PCR assay using novel selective primers with current methods in terms of ABO blood phenotyping in rhesus macaques

**DOI:** 10.1038/s41598-018-20395-0

**Published:** 2018-01-31

**Authors:** Yun-Jung Choi, Rae Hyung Ryu, Hye-Jin Park, Jae-Il Lee

**Affiliations:** 1 Graduate Course of Translational Medicine, Seoul National University College of Medicine, Seoul, 03080 Republic of Korea; 2 Transplantation Research Institute, Seoul National University Medical Research Center, Seoul, 03080 Republic of Korea; 3 Department of Medicine, Seoul National University College of Medicine, Seoul, 03080 Republic of Korea

## Abstract

Nonhuman primates are important animal models in transplantation. To prevent fatal transplantation-induced immune responses, it is necessary to accurately phenotype the monkey ABH antigens, which are the same as those in humans but (unlike in humans) are not expressed on red blood cells (RBCs). We compared the ability of two established ABO-typing methods, namely, serological testing and immunohistochemistry (IHC), and our novel polymerase chain reaction (PCR)-based assay to type 66 rhesus monkeys. The serological test assessed the ability of monkey sera to hemagglutinate human RBCs. The IHC assay measured the binding of murine anti-A and anti-B antibodies to monkey buccal mucosa cells. The whole blood-based PCR assay involved selective primers that were derived from the exon 7 sequences of A+, B+, and O+ monkeys. IHC and PCR unequivocally yielded the same types in all monkeys. Serological testing yielded inconsistent types in seven (10.6%). FACS analysis with monkey sera preabsorbed with O+ RBCs showed that the incorrect serological results related to nonspecific or xenoreactive binding of the human RBCs. Unlike previous PCR-based assay, our algorithm directly detected O+ monkeys and A and B homozygotes and heterozygotes. Given the logistical limitations of IHC, this PCR assay may be useful for typing rhesus monkeys.

## Introduction

To prevent fatal immunological reactions after organ transplantation in humans, it is essential that the organ donor and recipient are compatible in terms of ABO blood group^1–3^. Ensuring donor and recipient ABO blood group compatibility is also important when using experimental animal models of transplantation to evaluate potential immunotherapeutic strategies^4–6^. This is particularly true for models with nonhuman primates, which express the same ABH specificities of the human ABO blood group system^7^.

Unlike humans, however, most Old and New World monkeys do not express, or only weakly express, agglutinable A and B antigens on their red blood cell (RBC) surface^8–10^. This means that the standard RBC agglutination test cannot be used to determine the ABO type of these animals. To overcome this problem, other methods that detect A and B antigens in the saliva or the anti-A and anti-B antibodies in the serum have been introduced. These methods include the hemagglutination inhibition technique^10^, the saline agglutination method^9^, and the reverse gel system assay^11^. However, while these tools are simple and are often used, they can be prone to false results because of nonspecific binding to the human A+ or B+ RBC reagents by monkey anti-human heteroagglutinins^11^. Therefore, these tests cannot be used on their own for nonhuman primate ABO phenotyping.

A more objective and accurate method of typing nonhuman primates is to perform immunohistochemistry (IHC) on vascularized organs (*e.g*., the kidneys) and/or buccal mucosal cells^7,11^. When correctly performed, this method provides unequivocal results regarding blood group antigen expression^7^. However, its accuracy does depend on the level of blood group antigen expression by the epithelium, which can differ between subjects^12^, and the quality of the cells collected from epithelial tissues such as the buccal mucosa^13^. Moreover, it is difficult to keep epithelial cells fresh over a long period, unlike serum or DNA. In addition, nonhuman primates must be sedated to obtain buccal mucosa cells.

One method that could overcome the accuracy, technical, and logistical limitations of serological and IHC tests is polymerase chain reaction (PCR) determination of A−, B−, and O− specific single nucleotide polymorphisms (SNPs) in the *ABO* gene^14,15^. However, the PCR-based tools that have been reported to date are limited in terms of detecting the O phenotype in macaque monkeys. In fact, although Yamamoto *et al*. reported the presence of a mutation in exon 6 of *ABO* that would render the gene unable to produce a functional enzyme (although they did not specify its exact location)^16^ and other studies have shown by serological tests that up to 28% of macaques are O+^17^, three other PCR-based studies failed to detect any O+ macaques regardless of whether they used novel PCR techniques, IHC, or serological methods^13–15^. Thus, it remains unclear whether O-type macaque monkeys exist or whether the existing PCR methods cannot detect O alleles in macaque monkeys.

In the present study, we developed a PCR-based algorithm that utilizes novel primers that can discriminate between A, B, and O alleles in rhesus monkeys and can determine whether a monkey is homozygous or heterozygous for these alleles. This method was compared with a serological test and IHC to determine the accuracy with which it ABO-types rhesus monkeys. The relative limitations of serological tests and IHC for macaque typing are discussed.

## Materials and Methods

### Subjects

In total, 66 adult rhesus monkeys (*Macaca mulatta*) (29 males and 37 females) aged between 4 and 10 years and weighing between 3.9 and 8.5 kg were used in this study. All monkeys were obtained from Gaoyao Kangda Laboratory Animal Sciences & Technology Co., Ltd. (Guangdong, China) and were maintained at the Seoul National University Hospital (SNUH) Non-human Primate Center, which is an AAALAC-accredited facility. All animals used in this study were cared for in strict accordance with the National Institutes of Health Guide for the Care and Use of Laboratory Animals. This study was approved by the IACUC of SNUH (no. 14-0002-C2A0).

### Hemagglutination assay

The hemagglutination assay was conducted with the 66 monkey test sera and human RBC reagents as described previously^7^. Briefly, blood samples were collected from humans known to have A, B, or O blood type. The RBCs were separated by centrifugation at 2500 rpm for 10 minutes. After washing with phosphate-buffered saline (PBS, Sigma, St. Louis, MO, USA), the RBC suspension was centrifuged again at 2500 rpm for 10 minutes. The supernatant was discarded. This process was repeated twice. Finally, the RBCs were diluted to 5% with PBS. Monkey serum was obtained by taking whole blood from the femoral vein of the test animals, centrifuging it at 3500 rpm for 10 minutes, and collecting the serum. The serum was diluted to 10% with PBS. The diluted monkey serum was then mixed 2:1 with the human A+, B+, or O+ RBC suspensions and incubated at room temperature for 30 minutes. The agglutination response was observed both in the plate and under a microscope. The agglutination was graded as strong (+++), intermediate (++), weak (+), and negative (−).

### Flow cytometry analysis

In some cases, the agglutination reactions described above yielded discrepant results when compared with the results of other ABO-typing assays. Therefore, all 66 monkeys were subjected to confirmatory FACS analysis using flow cytometry. Thus, the monkey sera were mixed 2:1 with the human RBCs as described above, incubated for 30 minutes, and centrifuged at 1500 rpm for 5 minutes. The supernatant was removed, and the mixtures were washed twice. The samples were then stained with fluorescein isothiocyanate (FITC)-labeled goat anti-monkey IgG (Santa Cruz Biotechnology, Inc., TX, USA) at 4 °C for 30 minutes in the dark. After centrifugation at 1500 rpm for 5 minutes, the supernatant was removed. The mixtures were washed twice. The pellet was finally resuspended in 200 μl of PBS and subjected to flow cytometry on a FACS Calibur (Becton Bioscience, Mountain View, CA, USA). All data were analyzed using the FlowJo software (TreeStar, Ashland, OR, USA).

Due to discrepant cases, all monkey sera were also preabsorbed by human O+ RBCs and then subjected to FACS analysis. Thus, 5% human O+ RBCs were mixed 2:1 with 100 μl of 10% monkey serum, incubated at 37 °C for 30 minutes, and centrifuged at 1500 rpm for 30 minutes at 4 °C. The absorbed serum was taken, mixed 2:1 with the human A+, B+, and O+ RBCs, and prepared for FACs as described above.

### IHC staining

The ABH phenotyping of buccal mucosal smears was accomplished by IHC, as described previously^7^. Briefly, epithelial cells were collected by swabbing the surface of the buccal mucosa of the 66 rhesus monkeys with a sterile cotton-tipped applicator. Smears from each animal were applied to a microscope slide. The slides were fixed in cold acetone (Sigma, St Louis, MO, USA) for 10 minutes, then dried and washed with PBS. The respective 1:50 and 1:20 dilutions of murine anti-A and anti-B antibodies (Sigma, St. Louis, MO, USA) in PBS were prepared and added to the samples. After incubation at room temperature for 60 minutes and then washing with PBS, the slides were further incubated at room temperature for 60 minutes in the dark with 100 µl of 1:100-diluted FITC-conjugated goat anti-mouse IgM (Vector, Burlingame, CA, USA). The slides were washed in PBS and mounted using Antifade-DAPI (Vector)-1-6-Diamidino-2-phenylindole and stored in the dark at 4 °C. Staining was observed using a fluorescent microscope.

### Generation of novel selective primers and establishment of a PCR-based ABO-typing tool

To generate the novel primers and then use them to determine the ABO types of the test monkeys, the genomic DNA of the 66 monkeys was extracted from the whole blood using the QIAmp^®^ DNA Blood Mini Kit purchased from QIAGEN (Valencia, CA, USA). To amplify exon 7 of the *ABO* locus, PCR was conducted using the 5′ CCT GCC TTG CAG ATA CGT G 3′ (forward) and 5′ CAG CTG ATC ACG GGT TCC 3′ (reverse) primers, as reported previously^14^. PCR was performed in a Peltier Thermal Cycler (PTC)-200 with denaturation at 95 °C for 5 minutes followed by 35 cycles of 95 °C for 20 sec, 58 °C for 20 sec, and 68 °C for 1 minute. The PCR products were given a poly(A) tail and then inserted into the TA cloning vector pGEM-T Easy (Promega, Madison, WI, USA). The subcloned plasmid was prepared with an AccuPrep Plasmid Mini Extraction Kit (Bioneer, Daejeon, Korea) for sequence determination. The nucleotide sequence was confirmed with an ABI PRISM^®^ System (model 377). All other chemical reagents were purchased from Duchefa Biochemie (Haarlem, Netherlands).

To design a novel selective primer, genomic DNA was obtained from 10 of the 66 monkeys that had undergone ABO typing by serotyping and IHC staining. Their full exon 7 sequences (Supplementary Figure 1) were obtained as described above, and the SNPs in their ABO locus were identified. Two primer sets were selected for determining monkey ABO types. SET-1 consisted of the PA2-F, PO2-F, and CAB primers, while SET-2 consisted of the PB-F, PO3-F, and CAB primers (Table 1). To determine the ABO phenotype of the 66 monkeys, PCR was performed with the novel selective primers using Takara taq polymerase (Takara, Seoul, Korea). The PCR was performed in a PTC-200 with denaturation at 95 °C for 1 minute, followed by 30 cycles of 95 °C for 30 sec, 64 °C for 30 sec, and 72 °C for 30 sec. The PCR products were then subjected to electrophoresis in a 2% agarose gel.

**Table 1:** Primer sequences used in the PCR analyses.

Primer	SET	Sequences
E7F		*5*′*-CCTGCCTTGCAGATACGTG-3*′
E7R		*5*′*-CAGCTGATCACGGGTTCC-3*′
PA2-F	SET-1	*5*′*-TGGACGTGGACATGGAGTTCCTT-3*′
PO2-F		*5*′*-AGCCGGGAGGCCTTCACCTGC-3*′
CAB		*5*′*-CCCTGGTGAGCCGCTGCACCTCC-3*′
PB-F	SET-2	*5*′*-GGAGATCCTGACTCCGCTCTT-3*′
PO3-F		*5*′*-GCCACCGGGTCCACTACTTT-3*′
CAB		*5*′*-CCCTGGTGAGCCGCTGCACCTCC-3*′

E7F, exon 7 forward; E7R, exon 7 reverse; CAB, common AB reverse primer.

### Data availability

The data are available.

## Results

### Rhesus monkey ABO type frequencies

The ABO type of 66 adult rhesus monkeys was determined using a hemagglutination assay, IHC, and the PCR assay with the novel primers. The results are shown in Table 2. The IHC staining results were entirely consistent with the PCR results. These two tests showed that type B was most common (65%), followed by AB (18%), A (9%), and O (8%). Serotyping using the hemagglutination assay differed from the IHC and PCR findings in seven monkeys (10.6% of all monkeys) (Table 2). Therefore, if the blood phenotyping was determined by serotyping alone, the results may have affected the frequency of the different blood types.

**Table 2:** Results of ABO phenotyping in 66 rhesus monkeys using serotyping, immunohistochemistry, and PCR analysis.

ID	Serotyping		IHC staining	PCR analysis	ID	Serotyping		IHC staining	PCR analysis
	Microscope	Plate				Microscope	Plate		
R014*	**O**	**B**	**B**	**BB**	R153	B	B	B	BB
R021	B	B	B	BB	R154*	**B**	**B**	**AB**	**AB**
R022	AB	AB	AB	AB	R155	A	A	A	AO
R023	B	B	B	BO	R156	B	B	B	BB
R024	AB	AB	AB	AB	R195	B	B	B	BB
R039	O	O	O	OO	R197	B	B	B	BB
R054	B	B	B	BO	R199	B	B	B	BB
R055	B	B	B	BB	R200	B	B	B	BO
R056	AB	AB	AB	AB	R202	B	B	B	BB
R057	AB	AB	AB	AB	R203	AB	AB	AB	AB
R058*	**A**	**A**	**AB**	**AB**	R205	B	B	B	BB
R059*	**O**	**B**	**B**	**BB**	R207	B	B	B	BB
R060	B	B	B	BB	R209	O	O	O	OO
R061	O	O	O	OO	R1043	AB	AB	AB	AB
R062	A	A	A	AA	R1051	B	B	B	BB
R063	B	B	B	BB	R1079	B	B	B	BO
R064	B	B	B	BB	R1087	B	B	B	BO
R065*	**O**	**B**	**B**	**BB**	R1099	B	B	B	BB
R066	B	B	B	BB	R1105	AB	AB	AB	AB
R071	B	B	B	BO	R1109	AB	AB	AB	AB
R072	B	B	B	BB	R1121	B	B	B	BO
R073	B	B	B	BB	R1123	B	B	B	BB
R075	B	B	B	BB	R1165	O	O	O	OO
R076*	**AB**	**AB**	**A**	**AA**	R1171	B	B	B	BB
R081	AB	AB	AB	AB	R1187	B	B	B	BO
R083	O	O	O	OO	R1195	B	B	B	BB
R085	B	B	B	BO	R1203	B	B	B	BB
R133	B	B	B	BO	R1305	A	A	A	AO
R134	B	B	B	BB	R1365	B	B	B	BO
R135	B	B	B	BB	R1375	AB	AB	AB	AB
R136	B	B	B	BB	R1499	A	A	A	AO
R137	B	B	B	BB	R1551	B	B	B	BO
R152*	**AB**	**AB**	**A**	**AA**	R1973	B	B	B	BB

An asterisk (*) indicates inconsistency between the methods in terms of the monkey’s blood phenotype.

### Serotyping using hemagglutination response

The agglutination response of the 66 monkey sera was assessed both under a microscope and by macroscopic examination of the plate. Most samples exhibited distinct positive or negative aggregation, both under the microscope and in the plate (data not shown). However, seven monkeys showed confusing results such as weak or mixed-field agglutination. As a result, the microscope and plate serological phenotyping results in these monkeys were inconsistent with each other or partially inconsistent with the IHC and PCR results (Table 2). Specifically, in three monkeys (R014, R059, and R065), the plate results correctly indicated a B+ type whereas the microscope result incorrectly indicated an O type. In two monkeys (R058 and R154), the microscope and plate results both indicated an A or B type when the IHC and PCR results indicated an AB type. In the remaining two monkeys (R076 and R152), the reverse occurred: the microscope and plate results both indicated an AB type whereas the IHC and PCR results indicated an A type.

### FACS analysis of hemagglutinated serum samples

**Figure 1:**
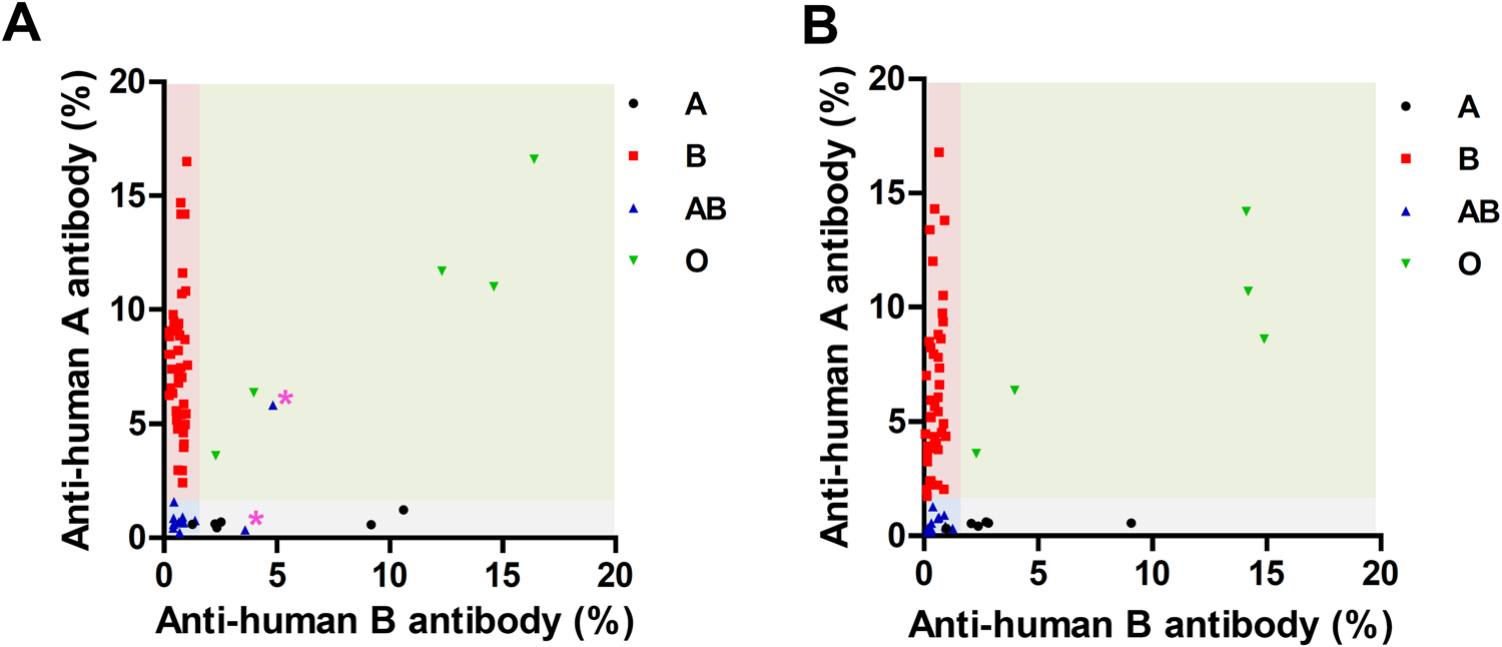
FACS analysis of the 66 rhesus monkey sera before (**A**) and after (**B**) preabsorption with human O+ red blood cells (RBCs). (**A**) Two monkeys (asterisks; R058 and R154) showed false-positive antibody binding to human A+ and B+ RBCs. (**B**) After preabsorption with human O+ RBCs, such nonspecific or xenoreactive binding was eliminated and the phenotypes of these two monkeys were corrected.

To determine whether the discrepant agglutination results were due to the presence of unknown nonspecific antibodies or xenoreactivity, the monkey serum:human RBC mixtures used in the agglutination assay were subjected to FACS analysis with a FITC-labeled anti-monkey antibody. The FACS results were consistent with the IHC and PCR results in 64 of the 66 monkeys, including in five of the seven monkeys that yielded inconsistent agglutination assay results. The remaining two monkeys (R058 and R154), whose respective microscope and plate results both indicated an A and a B type, respectively, when IHC and PCR indicated an AB type, exhibited nonspecific antibody binding: one erroneously exhibited an O type while the other exhibited an A type (asterisks, Fig. 1A). However, when these discrepant serum samples were subjected to preabsorption with human O+ RBCs and then FACS, the nonspecific binding (probably against unknown agglutinins) was clearly resolved: both displayed AB types (Fig. 1B).

Analysis of the FACS data with the preabsorbed sera of the 66 monkeys (Fig. 1B) showed that the type A and B monkey sera bound to human B+ and A+ RBCs, respectively, with at least 1.5% antibody binding. The five type O+ monkey sera bound strongly to both A+ and B+ RBCs. By contrast, the type AB+ sera recognized the A+ and B+ RBCs poorly. An exception was the R152 monkey, which was incorrectly typed as AB+ by the agglutination assay and typed as A+ by IHC and PCR: its serum bound only relatively weakly to B+ RBCs (0.97%).

### ABO typing by IHC staining

**Figure 2:**
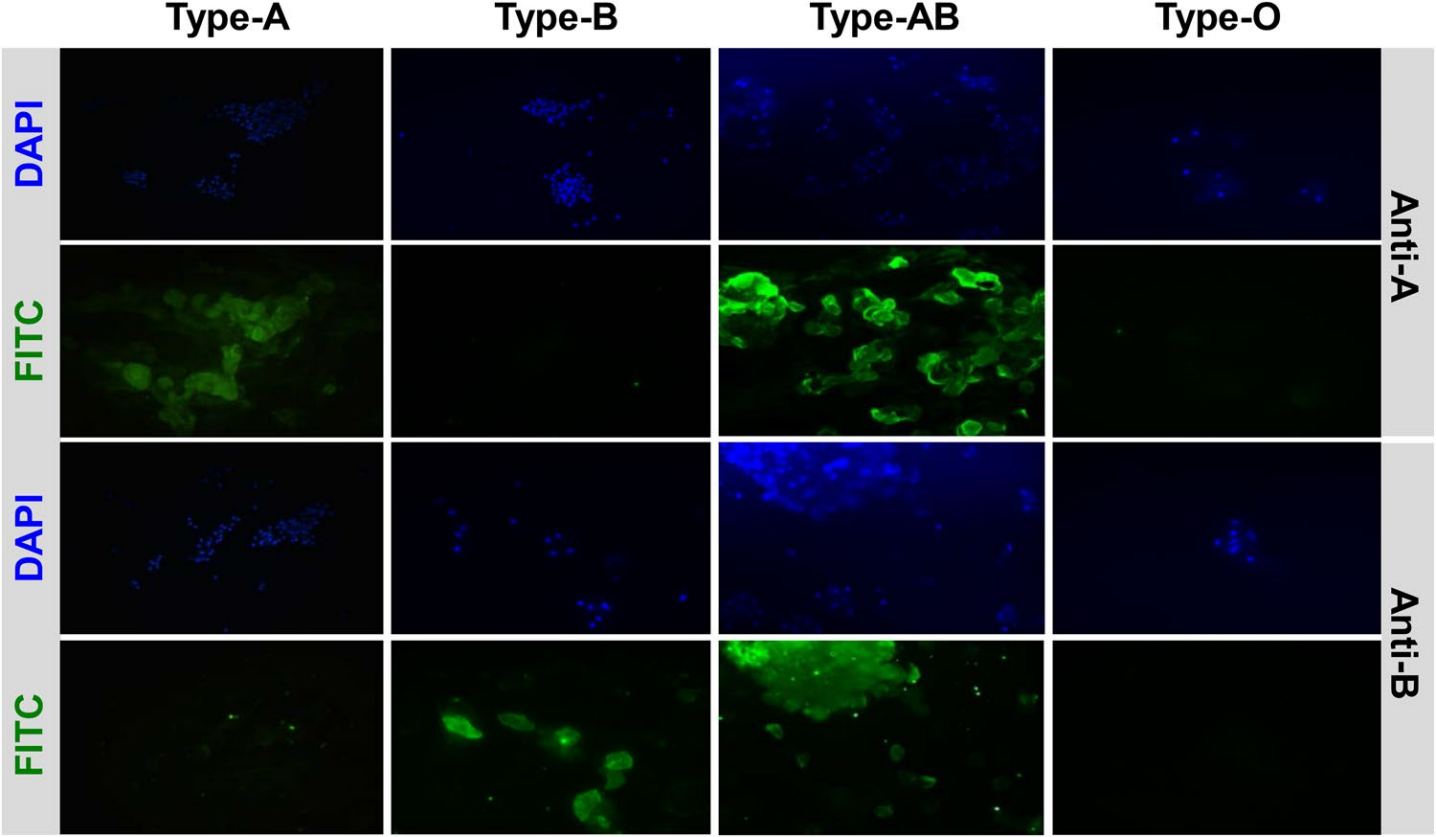
Immunohistochemical staining of buccal mucosal cells from the rhesus monkeys. Representative images are shown. The cells from A+ and B+ monkeys were recognized by FITC-labeled murine anti-A and anti-B antibodies, respectively. The cells from AB+ monkeys were recognized by both murine antibodies. The cells from the O+ monkeys did not react with either antibody. DAPI, 4′,6-diamidino-2-phenylindole; FITC, fluorescein isothiocyanate.

IHC staining of the buccal mucosal cells with anti-A and anti-B antibodies clearly identified the blood phenotypes in all monkeys: all twice smears taken from each animal yielded consistent results (Fig. 2). Although impurities in the mucosal samples could not be entirely removed at the staining process, they did not interfere with the determination of blood type.

### Determination of the ABO alleles by a PCR assay

**Figure 3:**
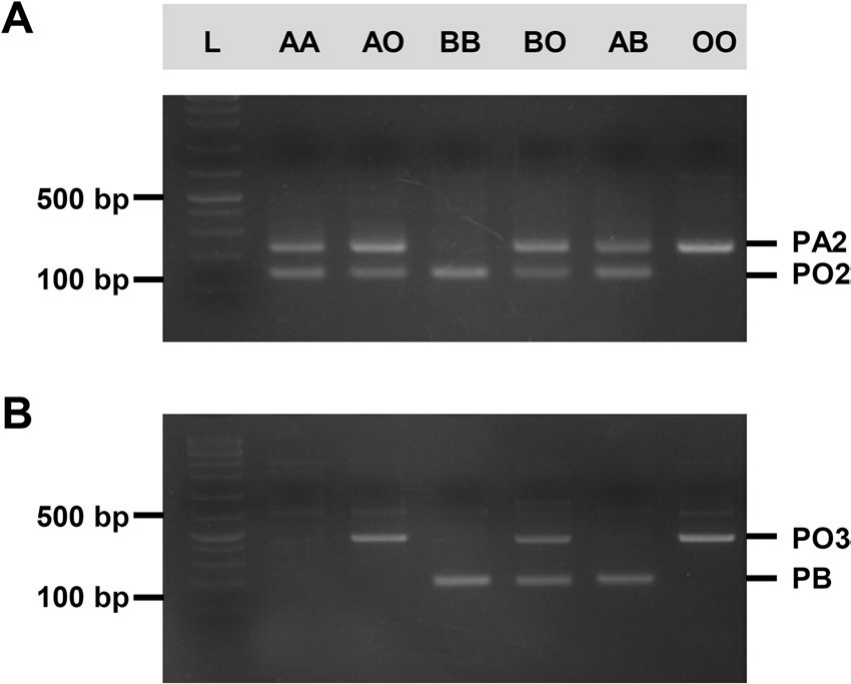
PCR banding patterns for the AA, AO, BB, BO, AB, and OO blood phenotypes in rhesus monkeys. Two ABO allele-specific primer sets were generated on the basis of single nucleotide polymorphisms in exon 7 of the *ABO* locus. Whole blood from 66 rhesus monkeys was subjected to PCR with the primer. (**A**) SET-1 consisted of PA2 and PO2. (**B**) SET-2 consisted of PO3 and PB. The SET-1- and SET-2-based PCR algorithm successfully determines homozygosity and heterozygosity for A, B, and O. AA homozygotes are indicated by the PA2 and PO2 bands, AO heterozygotes are indicated by the PA2, PO2, and PO3 bands, BB homozygotes are indicated by the PO2 and PB bands, BO heterozygotes are indicated by all four bands, AB heterozygotes are indicated by the PA2, PO2, and PB bands, and OO homozygotes are indicated by the PA2 and PO3 bands. L, ladder; bp, base pair.

On PCR with the novel selective-designed primer sets, AA homozygotes were indicated by the PA2 and PO2 bands, AO heterozygotes were indicated by the PA2, PO2, and PO3 bands, BB homozygotes were indicated by the PO2 and PB bands, BO heterozygotes were indicated by all four bands, and AB types were indicated by the PA2, PO2, and PB bands. Significantly, our algorithm was also able to detect O homozygotes: they were indicated by the PA2 and PO3 bands (Fig. 3). In other words, absence of PO2 band that exhibited by G → C SNP (nucleotide positions 5196271) was significant in defining the O homozygotes. This is important because previously reported PCR assays have not been able to detect O homozygotes in macaque monkeys^8,13,14^. All of our PCR results were entirely consistent with the IHC results with buccal mucosal cells. Notably, our PCR assay has an advantage over all other serotyping methods tested here, even IHC: it can determine whether individual monkeys are heterozygotes or homozygotes for the A and B alleles.

## Discussion

The ABH antigens are the representative alloantigen system in mammals. Various commonly used ABO phenotyping assays, namely, a serological test and IHC staining, were compared with a new PCR-based tool in this study. While the IHC and PCR assays yielded clear and identical typing results, the serological test did not: it yielded types in 7 of 66 monkeys (10.6%) that were discrepant from the IHC and PCR results.

The serological test we used in the present study measured the hemagglutination response of monkey sera to human A+, B+, and O+ RBCs. It is a very simple and very well-known method that was established long ago. However, it has been found repeatedly to occasionally yield incorrect types when it is compared with other typing methods. For example, Chen *et al*. showed that, when they compared the reverse gel test using untreated sera from rhesus monkeys with IHC, there were confusing or incorrect results in 15.8% of cases^11^. This is similar to our rate of false results (7/66; 10.6%).

We asked whether FACs analysis, which has not yet been used for ABO typing, could correctly type the 66 monkey samples, including the seven samples that were typed incorrectly by the serological test. Thus, we washed the monkey serum:RBC mixtures, incubated them with a FITC-labeled anti-monkey antibody, and performed FACS analysis. The FACs results were unequivocally consistent with the IHC and PCR results in all but 2 of the 66 samples: those two samples had also been mistyped by the serological test. However, when we preabsorbed the 66 monkey samples with human O+ RBCs, all samples, including the two discrepant ones, were typed correctly (Fig. 1). This suggests that the discrepant FACS results were due to interference caused by nonspecific binding, possibly by unknown nonspecific antibodies or xenoreactive antibodies in the monkey sera. These findings suggest that serological tests should only be performed with sera that have been preabsorbed by human O+ RBCs. It should be noted, however, that, even after such treatment of the sera, the FACS data were not all unequivocal: one type A monkey exhibited only weak binding to B+ RBCs. Thus, FACS-based ABO tests may also be subject to some degree to equivocal or incorrect typing.

Two studies have reported that IHC on buccal mucosal smears or renal tissues yields relatively consistent and accurate blood types in baboons, cynomolgus monkeys, and pigs^7,11^. Recently, however, Wang *et al*. warned that IHC of buccal mucosal cells should serve as a supplementary method rather than as a gold standard because its accuracy can be compromised by inadequate sample preparation that results in insufficient buccal mucosal cells or the presence of impurities^18^. In the present study, we found that IHC staining of buccal mucosal cells was more consistently unequivocal and more accurate than serotyping. However, the method does have some logistical and technical disadvantages. First, it is necessary to chemically immobilize the monkeys to obtain the buccal mucosa samples. Second, the sample must be fresh. Third, there can be nonspecific binding of the mouse anti-human A and B antibodies and the FITC-conjugated goat anti-mouse IgM, which can yield false-positive or false-negative results.

Premasuthan *et al*. and Kim *et al*. recently introduced PCR assays for ABO typing rhesus and cynomolgus monkeys^13–15^. However, these methods only detected A, B, and AB phenotypes. Moreover, while the O phenotype of humans associates with a mutation that introduces a stop codon in exon 6 that prevents the addition of polysaccharides to the H antigen^16,19–21^, several studies failed to detect similar functional mutations in exon 6 that are responsible for the O phenotype in macaques^8,13,14^. These observations have led to speculation that macaques lack the O phenotype. However, previous reports based on serological testing and IHC staining^7,18^ demonstrated the presence of the O phenotype in macaques. Thus, a PCR assay that can directly identify the O+ phenotype in macaque monkeys would be valuable.

To address this, we sequenced exon 7 of the *ABO* locus of 10 monkeys whose ABO phenotype had been determined by serotyping and IHC. One was an O+ monkey. On the basis of the *ABO* exon 7 sequences of these monkeys, we designed novel selective PCR primers that when used together in a novel algorithm were able to distinguish between the A and O phenotypes (Fig. 3). Presumably, it seems that nonsense mutation due to C → G SNP is result in creating an O phenotype in rhesus monkeys. This assay was demonstrated to be valid because all PCR-determined blood phenotypes corresponded exactly with the IHC phenotypes (Table 2). Thus, PCR analysis using our primer sets can detect the O phenotype and can serve as an accurate screening test for ABO blood type in rhesus monkeys.

In summary, serological testing for hemagglutination responses is limited by occasional interference from nonspecific antibodies in the monkey serum. Thus, this method is not entirely reliable. IHC-based typing of buccal mucosa cells provided unequivocal typing results but the method associates with logistical limitations and requires careful sample preparation. By contrast, our PCR-based assay on whole blood is simple and provides unequivocal results that are entirely consistent with the IHC results. Moreover, it identifies O+ specimens directly, whereas on the serological and IHC assays, this type is only inferred on the basis of the absence of A and B specificities.

## Electronic supplementary material

Supplementary information
