# Supplementary material for: Comparison of a PCR assay using novel selective primers with current methods in terms of ABO blood phenotyping in rhesus macaques
Source: Sci Rep. 2018 Jan 31;8:1957. doi: 10.1038/s41598-018-20395-0 (PMC5792491; doi:10.1038/s41598-018-20395-0)
Supplement: Supplementary file 1 — Supplementary information [file 41598_2018_20395_MOESM1_ESM.pdf]

Comparison of a PCR assay using novel selective primers with current methods in terms of ABO blood phenotyping in rhesus macaques

Yun-Jung Choi[1], Rae Hyung Ryu[2], Hye-Jin Park[2], Jae-Il Lee[2,3*]

[1]Graduate Course of Translational Medicine, Seoul National University College of Medicine, Seoul 03080, Republic of Korea
[2]Transplantation Research Institute, Seoul National University Medical Research Center, Seoul 03080, Republic of Korea
[3]Department of Medicine, Seoul National University College of Medicine, Seoul 03080, Republic of Korea

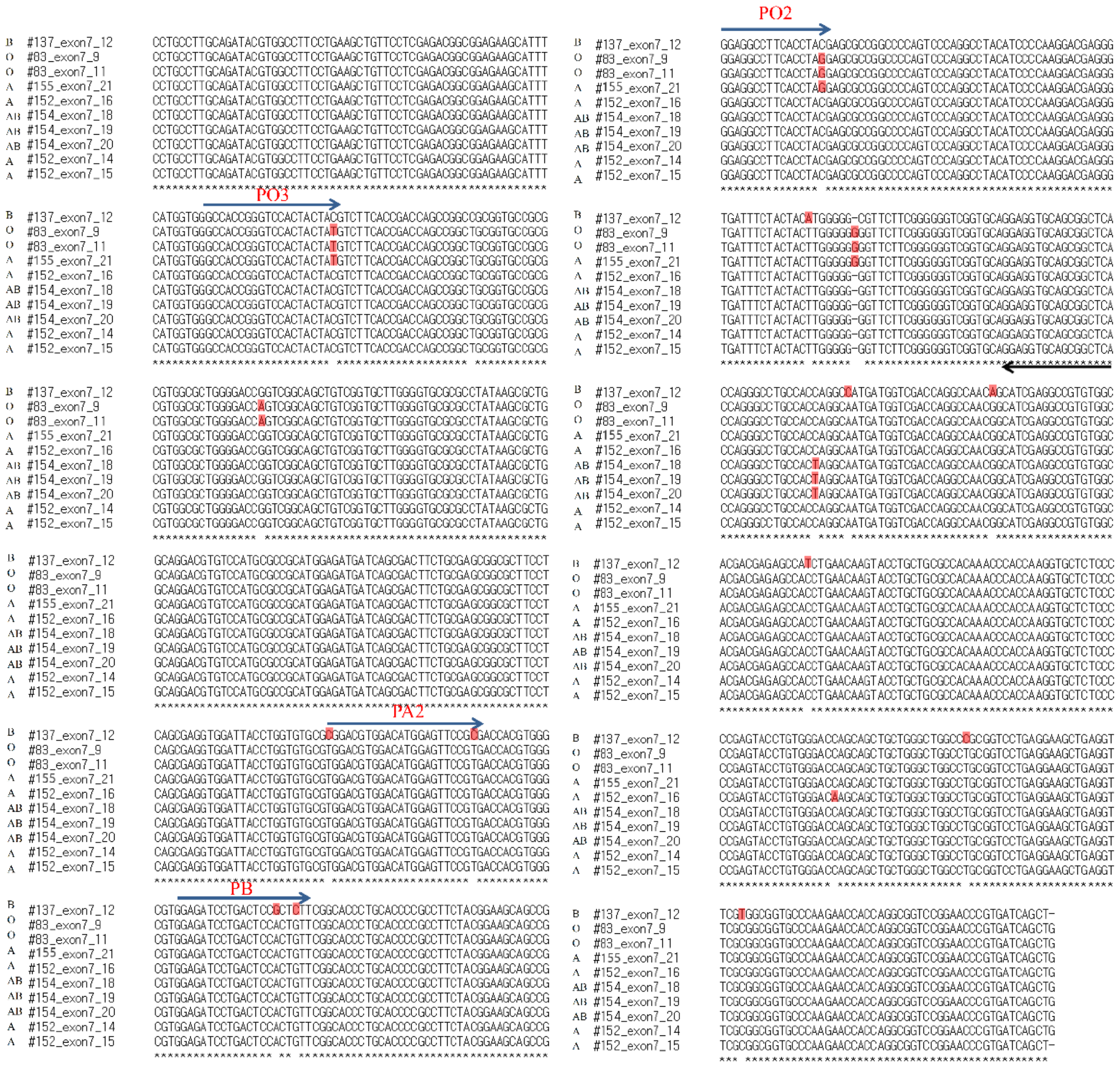

Figure S1. Full exon 7 sequences were obtained from chromosome 15 of rhesus monkeys. The red highlights indicate single nucleotide polymorphisms, the blue arrows indicate forward primer sequences (PO3, PA2, PB, and PO2), and the black reverse arrow indicates a reverse primer sequence (CAB).
